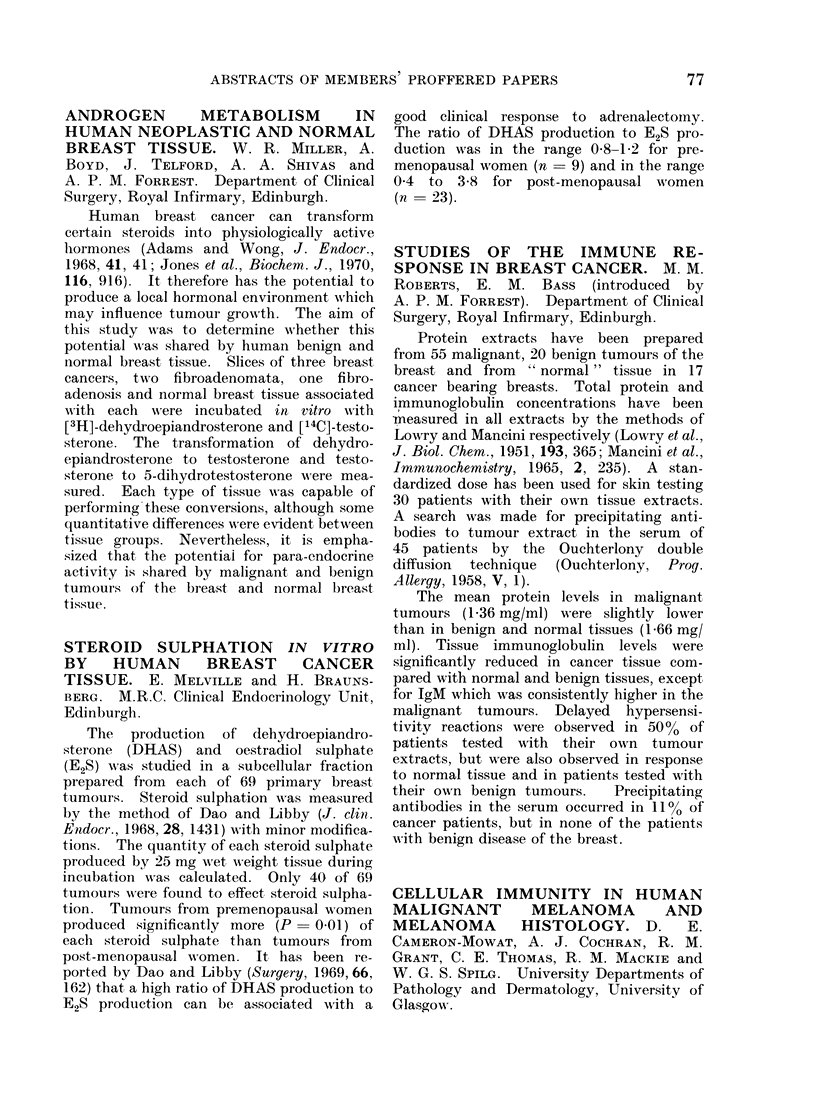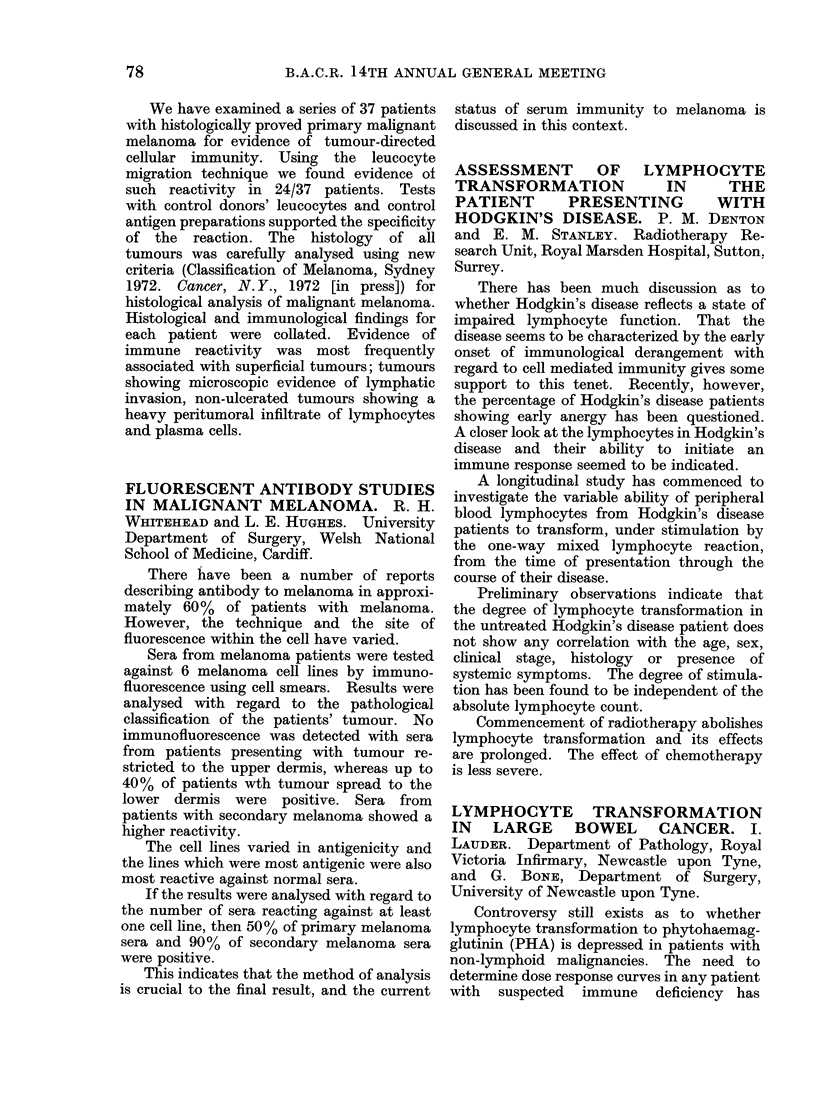# Cellular immunity in human malignant melanoma and melanoma histology.

**DOI:** 10.1038/bjc.1973.79

**Published:** 1973-07

**Authors:** D. E. Cameron-Mowat, A. J. Cochran, R. M. Grant, C. E. Thomas, R. M. Mackie, W. G. Spilg


**CELLULAR IMMUNITY IN HUMAN MALIGNANT MELANOMA AND MELANOMA HISTOLOGY.** D. E. CAMERON-MOWAT, A. J. COCHRAN, R. M. GRANT, C. E. THOMAS, R. M. MACKIE and W. G. S. SPILG. University Departments of Pathology and Dermatology, University of Glasgow.

We have examined a series of 37 patients with histologically proved primary malignant melanoma for evidence of tumour-directed cellular immunity. Using the leucocyte migration technique we found evidence of such reactivity in 24/37 patients. Tests with control donors' leucocytes and control antigen preparations supported the specificity of the reaction. The histology of all tumours was carefully analysed using new criteria (Classification of Melanoma, Sydney 1972. *Cancer, N.Y.*, 1972 [in press]) for histological analysis of malignant melanoma. Histological and immunological findings for each patient were collated. Evidence of immune reactivity was most frequently associated with superficial tumours; tumours showing microscopic evidence of lymphatic invasion, non-ulcerated tumours showing a heavy peritumoral infiltrate of lymphocytes and plasma cells.